# Prevalence and factors associated with musculoskeletal disorders among primary and secondary school teachers

**DOI:** 10.3389/fpubh.2025.1654131

**Published:** 2025-10-28

**Authors:** John Marwa Gikaro, George Claud Goi, Farida Hassan Taamala, Hussein Hamisi Hamadi, Jenifa Charles Welema, Zakia Mussa Minduva, Elia Asanterabi Swai

**Affiliations:** ^1^Department of Physiotherapy, School of Rehabilitation Medicine, KCMC University, Moshi, Tanzania; ^2^Department of Physiotherapy, Kilimanjaro College of Health and Allied Sciences, Moshi, Tanzania; ^3^Department of Epidemiology and Global Health, Umeå University, Umeå, Sweden; ^4^Department of Community Medicine and Rehabilitation, Umeå University, Umeå, Sweden

**Keywords:** work-related musculoskeletal disorders, occupational health, teachers, Tanzania, Moshi municipality

## Abstract

**Background:**

Musculoskeletal disorders (MSDs) are among the top conditions that affect the working population including teachers. Historically, Kilimanjaro region including Moshi municipal, has been a pioneer in education development in Tanzania. Moshi municipality represent a diverse setting of primary and secondary education; however, no study has yet examined MSDs among teachers in this setting.

**Objective:**

The aim of this study was to investigate the prevalence of MSDs among primary and secondary school teachers, and identify the associated factors.

**Methodology:**

A cross-sectional study was conducted among 205 primary and secondary school teachers in Moshi Municipal, Northern Tanzania. Data was collected using a structured questionnaire that incorporated the Standardized Nordic Musculoskeletal Questionnaire (SNMQ). Logistic regression model was used to estimate the factors associated with MSDs.

**Results:**

The 12-month and 7-day prevalence of MSDs in one or more body sites was 61.5% and 44.4%, respectively. Twelve-month prevalence was higher in the lower back (44.4%), followed by the neck (31.2%), upper back (26.8%), and shoulders (18.5%). Seven-day prevalence was higher in the lower back (29.8%), followed by the neck (16.1%), ankles/feet (12.2%), and upper back (9.8%). Predictors of MSDs at different body parts were the age, gender, BMI, working duration, number of working hours, number of classes, and number of students in class.

**Conclusion:**

Occupational factors significantly contribute to MSDs among teachers. Complying to recommended workload for teachers may help to prevent MSDs in teaching profession.

## Introduction

Musculoskeletal disorders (MSDs) are among the commonest conditions in the working population that is engaged in jobs that involve repetitive tasks, prolonged static or awkward postures, and manual handling (1). MSDs affect body structures such as muscles, tendons, ligaments, fascia, nerves and joints depending on their involvement with job tasks (2); for instance, a fault to a muscle or tendon may transpire because of overuse, or prolonged awkward posture. MSDs occur in highly mobile body parts such as the neck and back, weight bearing joints such as hips and knees, and major muscle groups such as paraspinals and shoulder stabilizers that maintain body posture during working (3).

The teaching profession, especially in lower levels, involves physical and psychological demands that make teachers highly vulnerable to MSDs (4). Teachers are required to maintain prolonged static postures such as standing or sitting during preparation of lessons, lecturing, or marking assignments (5, 6). Similarly, teachers are exposed to repetitive tasks such as writing on boards, grading papers, and when using assistive teaching equipment (7, 8). Low teacher-to-student ratio increases the workloads that have both physical and psychological demands. In general, prolonged postures lead to fatigue; repetitive tasks lead to microinjuries; poorly designed furniture at work, particularly in low resourced areas, lead to strain; and high workloads pose a significant risk for teachers to develop MSDs at work (4, 6, 9). Additionally, psychosocial work stressors of the profession exacerbate tension in major muscle involved in maintaining posture, which can increase the risk of developing MSDs (10). Treating teachers with MSDs has been proven to increase healthcare costs (4). MSDs can negatively influence active engagement in teaching tasks leading to decreased productivity (11). Moreover, absenteeism caused by MSDs is likely to interfere with the students’ learning environment potentially leading to reduced academic performance (12). Research shows that MSDs are among the top occupational hazards in teaching profession across the globe (13). In recent years, highest prevalence of MSDs among teachers have been recorded in Brazil (6), Chile (14), Taiwan (15), Saudi Arabia (16), Pakistan (17), Nigeria (18), and Cameroon (19). While, lower records have been reported in Turkey (20), Iran (21), and Sweden (22). Additionally, in Chile, the prevalence of MSDs among teachers in urban public school was slightly higher than that of teachers from rural areas (23). Tanzania, like many developing countries, faces unique challenges in its education, especially overcrowded classrooms, and poor infrastructure. However, there is limited scrutiny of potential contextual factors unique to teaching profession in Tanzania. As a result, little is known regarding the magnitude of the MSDs among teachers, associated factors, and intervention that aligns with the national health system.

Kilimanjaro region is one of the regions with highest number of primary and secondary schools in Tanzania. Moshi municipal alone, the administrative and economic capital of Kilimanjaro region, has a total of 80 primary and secondary schools. The introduction of the free primary and secondary education system has improved the number of pupils attending school; however, the teacher–student ratio and other occupational factors could influence teachers experience on MSDs, especially in a setting with diverse education settings like Moshi municipality. Studying teachers in Moshi municipality gives a reflection of teachers in the region, that may represent majority in the country. Our study aimed at exploring the prevalence and factors associated with MSDs among primary and secondary school teachers in Northern Tanzania. We believe that the insights obtained from this study could be useful in setting baseline prevention and intervention strategies for MSDs among primary and secondary teachers in the country.

## Materials and methods

### Study design and participants

This cross-sectional study was conducted from June to July 2024 in 14 public primary and secondary schools in Moshi Municipal, Kilimanjaro region, Tanzania. Teachers in respective schools who met inclusion criteria were included in the study. We included teachers from public schools. Exclusion criteria were working experience of less than 1 year, recent surgeries/trauma, and systemic conditions, for example systemic lupus erythromatosus and Rheumatoid arthritis, for they directly affect musculoskeletal system. Multi-stage sampling technique was used to obtain study participants. Researchers identified the 21 wards in Moshi Municipality, with a total of 80 schools (52 primary and 28 secondary schools). In the initial stage, 10 out of 21 wards were selected using simple random technique. All 31 schools from the 10 wards were listed using numerals 1–31. We included schools that happened to be assigned even numbers. Generally, 14 public schools (eight primary and six secondary schools) were systematically selected. Finally, simple random sampling was used to select participants from the selected schools. The total number of teachers in Moshi municipal at the time of data collection was 567. Cochran’s formula for finite population (24) was used to estimate sample size. The estimated sample size was 230, however, 205 teachers who were present during the time of data collection, and willing to participate in the study were recruited.

### Data collection

Data were collected using a self-reported structured questionnaire that incorporated the Standardized Nordic Musculoskeletal Questionnaire (SNMQ). The questionnaire had three parts: Part A regarding demographic characteristics, Part B inquiring about occupational factors, and Part C with information of body parts with MSDs according to SNMQ (25). Pilot study was done to validate the questionnaire prior to actual data collection. Ten teachers, five from primary school and five from secondary school, were conveniently recruited to participate in the pilot study. They were instructed to complete the questionnaire and provide feedback that was used to improve its clarity and validity. Data gathered during piloting was not included in final analysis. Participants were asked to fill in all relevant parts of the questionnaire and collect as soon as they finished. All participants provided written consent, and they were free to withdraw from the study any time they felt like so. All procedures were conducted according to the Helsinki Declaration. Permission to conduct this study was ratified by the College Research Ethics Review Committee (No. UG.132/2024), and Moshi Municipal Council (Ref. No. MMC/A.40/13/1VOL.33/332).

### Musculoskeletal disorders

The SNMQ was used for evaluating MSDs among teachers. The questionnaire provides information about pain prevalence in 12 body areas, that is, the neck, shoulders, elbows/forearms, wrists/hands, upper back, lower back, hips/thighs, knees, and feet/ankles. The tool inquires information about pain/discomfort/ache in any of the areas for the past 12 months, and 7 days. Also, the tool help to investigate whether there were limitations caused by the MSDs at home or work.

### Data analysis

Collected data were entered in Microsoft Excel. Data cleaning was done to check for errors during data entry. Data was then imported to R software for Windows (version 4.4.1) for further management and analysis. Findings for descriptive statistics were presented using frequencies, percentages, and chi-square test. Shapiro–Wilk normality test was used to determine the distribution of continuous variables. Continuous variables that were normally distributed were processed using means with respective standard deviations (M ± SD), non-normally distributed variables were presented using medians and respective interquartile ranges (IQR). Sociodemographic, anthropometric, and occupational factors among teachers were compared by gender. Prevalence of MSDs were presented as frequency and percentage according to presence in the previous 12 months and 7 days. To account for participants with MSDs in multiple body sites, grouping was done into percentile 50 according to the previous 12 months and 7 days, where in both cases p50 was 2. MSDs were compared with sociodemographic and occupational variables among teachers with MSDs in two painful regions or less (≤p50) or more than two painful regions (>p50). Physical activity level was classified according to recommendations of the World Health Organization: adequate, if the participant engaged in moderate-intensity physical activity for at least 150 min per week, and inadequate, if the time for moderate-intensity physical activity was less than 150 min in a week. Categorical variables were compared using the chi-squared and Fisher exact tests for the association. Backward elimination method, with *p*-value set at 0.05, was used to select the predictor factors that were suitable to fit the model. Logistic regression models were conducted to analyze the association between the MSDs (≤p50 and >p50) and selected factors with reference to absence of MSDs (no MSDs). The statistical significance was set at *p* < 0.05 (95% confidence interval). Finally, the Hosmer–Lemeshow test was used to evaluate the models’ goodness of fit, where values above 0.05 indicated that the model fit the data.

## Results

### Demographic characteristics

Shapiro test showed that data for BMI and age were not normally distributed (*p* < 0.05). Majority of participants were females (62%), and aged less than 40 years (50.2%) with median age of 39 years. The proportion of MSDs was high in females (66.1%) than in males (50%). For the past 12 month, 61.5% of the participants had MSDs in one or more body site, females leading. Similarly, over the past 7 days, 44.4% of participants had MSDs in one or more body sites. The proportion of females with limitations resulting from MSDs was nearly twice as males. The proportion of females who were obese was over two times higher (37%) than males (17.9%). Female teachers with certificate level of education were over four times (14.2%) compared to males (3.8%). The detailed participant characteristics is given in Table 1.

**Table 1:** Demographic characteristics stratified by gender.

Variable	Overall (*N* = 205)	Female (*N* = 127)	Male (*N* = 78)	*p*-value
	*N* (%)	%	%	
Age (median [IQR])	39.00 [36.00, 45.00]	39.00 [35.00, 45.00]	40.00 [36.00, 46.00]	0.201
Age group				0.293
< 40 years	103(50.2)	52.8	46.2	
40-50 years	79(38.5)	36.2	42.3	
> 50 years	23(11.2)	11.0	11.5	
BMI (median [IQR])	27.05 [24.65, 30.80]	28.30 [24.95, 32.00]	25.61 [23.40, 28.33]	<0.001
BMI group ranges				0.005
Underweight	3(1.5)	0.0	3.8	
Normal	61(29.8)	26.0	35.9	
Overweight	80(39.0)	37.0	42.3	
Obese	61(29.8)	37.0	17.9	
Education level				0.044
Certificate	21(10.2)	14.2	3.8	
Degree	122(59.5)	53.5	69.2	
Diploma	48(23.4)	26.0	19.2	
Master	14(6.8)	6.3	7.7	
Marital status				0.726
Divorced	2(1.0)	0.8	1.3	
Married	174(84.9)	84.3	85.9	
Single	23(11.2)	11.0	11.5	
Widowed	6(2.9)	3.9	1.3	
Smoking (Yes)	7(3.4)	0.0	9.0	0.002
Alcohol drinking (Yes)	51(24.9)	20.5	32.1	0.090
Work duration				0.441
<10 years	63 (30.7)	33.1	26.9	
≥ 10 years	142 (69.3)	66.9	73.1	
Work position				0.856
Combination of all positions	63(30.7)	29.9	32.1	
Sitting	12(5.9)	6.3	5.1	
Standing	122(59.5)	59.1	60.3	
Walking	8(3.9)	4.7	2.6	
Extracurricular activities (Yes)	137(66.8)	62.2	74.4	0.101
Commuting distance (mean (SD))	5.67 (5.95)	5.26 (5.99)	6.34 (5.87)	0.210
Physical activity				0.030
Adequate	23 (11.2)	7.1	17.9	
Inadequate	182 (88.8)	92.9	82.1	
12-month MSDs				0.056
>p50	58 (28.3)	32.3	21.8	
≤p50	68 (33.2)	35.4	29.5	
7-day MSDs				0.001
>p50	33 (16.1)	19.7	10.3	
≤p50	58 (28.3)	34.6	17.9	
Any limitation by MSDs				0.042
No	139 (67.8)	62.2	76.9	
Yes	66 (32.2)	37.2	23.1	

BMI, body mass index; IQR, interquartile range; KM, kilometer; MSDs, musculoskeletal disorders; *N*, number; SD, standard deviation.

### Prevalence of MSDs

For the past 12 months, MSDs were higher in the lower back (44.4%), followed by the neck (31.2%), upper back (26.8), and shoulders (18.5). Nearly three-quarters of participants with MSDs at the lower back (72.7%) presented with activity limitation at home or work. Correspondingly, more than half of participants with MSDs at neck (56.1%), and upper back (51.5%) were limited from normal work. For the past 7 days, MSDs were higher in the lower back (29.8%), followed by the neck (16.1%), ankles/feet (12.2%), upper back (9.8%), shoulders (9.8%), and knees (8.8%). Over half (53%) of participants with MSDs at the lower back were limited from normal work at home or away from home, while over a quarter of participants with MSDs at the ankle/feet (25.8%), and neck (28.8%) were limited from normal work. Prevalence of MSDs at each body site is provided in Table 2.

**Table 2:** Prevalence of MSDs in the past 12-months, and 7-days stratified by limitation from work.

Variable	Overall (*N* = 205)	Limited from work (*n* = 66)	Not limited from work (*n* = 139)	*p*-value
	*N* (%)	%	%	
12-months MSDs prevalence	126 (61.5)			
Neck (Yes)	64 (31.2)	56.1	19.4	<0.001
Shoulders (Yes)	38 (18.5)	34.8	10.8	<0.001
Elbow (Yes)	22 (10.7)	24.2	4.3	<0.001
Wrist/hands (Yes)	33 (16.1)	31.8	8.6	<0.001
Upper back (Yes)	55 (26.8)	51.5	15.1	<0.001
Lower back (Yes)	91 (44.4)	72.7	30.9	<0.001
Hips/thighs (Yes)	18 (8.8)	19.7	3.6	<0.001
Knees (Yes)	28 (13.7)	24.2	8.6	0.005
Ankles/feet (Yes)	25 (12.2)	27.3	5.0	<0.001
7-days MSDs prevalence	91 (44.4)			
Neck (Yes)	33 (16.1)	28.8	10.1	0.001
Shoulders (Yes)	20 (9.8)	9.7	5.0	0.002
Elbow (Yes)	10 (4.9)	6.1	4.3	0.846
Wrist/hands (Yes)	15 (7.3)	10.6	5.8	0.338
Upper back (Yes)	20 (9.8)	19.7	5.0	0.002
Lower back (Yes)	61 (29.8)	53.0	18.7	<0.001
Hips/thighs (Yes)	6 (2.9)	7.6	0.7	0.023
Knees (Yes)	18 (8.8)	15.2	5.8	0.05
Ankles/feet (Yes)	25 (12.2)	25.8	5.8	<0.001

MSDs, musculoskeletal disorders; *N*, Overall; *n*, number.

### Association between presence of MSDs and occupational factors

The logistic regression model for 12-month MSDs prevalence showed that there was a significant positive association between presence of MSDs in two or less body sites (≤p50) and BMI (OR = 1.14, *p* = 0.001), and number of working hours per day (OR = 1.175, *p* = 0.008). Additionally, MSDs in more than two body sites (>p50) was positively and significantly associated with BMI (OR = 1.19, *p* < 0.001), and number of classes (OR = 1.48, *p* = 0.047). Details of variables selected by backward elimination for the model, and odds ratios with respective 95% confidence intervals are shown in Table 3.

**Table 3:** Factors associated with 12-month MSDs prevalence.

Variable	No MSDs	MSDs ≤ p50		MSDs > p50	
	OR (95%CI)	OR (95%CI)	*p*-value	OR (95%CI)	*p*-value
BMI	Ref	1.14 (1.05–1.24)	0.001*	1.19 (1.09–1.30)	<0.001*
Work duration	Ref	1.02 (0.97–1.08)	0.490	1.06 (1.01–1.12)	0.024*
Number of work hours	Ref	1.75 (1.16–2.65)	0.008*	1.09 (0.72–1.65)	0.688
Number of classes	Ref	1.12 (0.77–1.62)	0.558	1.48 (1.01–2.18)	0.047*
Number of students in class	Ref	0.99 (0.99–1.00)	0.243	0.99 (0.99–1.00)	0.109
Number of breaks	Ref	0.37 (0.09–1.57)	0.178	1.44 (0.41–5.09)	0.573
Work position	Ref				
Standing	Ref	0.54 (0.25–1.20)	0.129	0.82 (0.34–1.93)	0.643
Sitting	Ref	0.49 (0.06–3.84)	0.496	4.67 (0.85–25.62)	0.076
Walking	Ref	1.89 (0.20–17.47)	0.576	4.49 (0.48–41.82)	0.188
Presence of assistive teaching devices (Yes)	Ref	0.52 (0.23–1.18)	0.118	0.87 (0.39–1.96)	0.738
Commuting distance	Ref	0.94 (0.88–1.00)	0.053	0.98 (0.92–1.04)	0.529
Hosmer–Lemeshow test (*p*-value)	0.8943				

OR, odds ratio; CI, confidence interval; MSDs, musculoskeletal disorders; p50, 50th percentile (*n* = 2); Ref, reference category. *p-value was significant.

The 7-day MSDs prevalence regression model revealed that there was a significant positive association between MSDs at two or less body sites (≤p50) and female gender (OR = 2.87, *p* = 0.008), and number of students in class (OR = 1.01, *p* = 0.023). On the other hand, MSDs at more than two body sites (>p50) was positively and significantly associated with age (OR = 1.08, *p* = 0.009), and BMI (OR = 1.12, *p* = 0.013). Variables retained during backward stepwise elimination method, odds ratios and respective 95% confidence intervals are shown in Table 4.

**Table 4:** Factors associated with 7-day MSDs prevalence.

Variable	No MSDs	MSDs ≤ p50		MSDs > p50	
	OR (95%CI)	OR (95%CI)	*p*-value	OR (95%CI)	*p*-value
Age	Ref	1.01 (0.96–1.06)	0.629	1.08 (1.02–1.14)	0.009*
Gender (female)	Ref	2.87 (1.31–6.30)	0.008*	2.50 (0.90–6.89)	0.078
BMI	Ref	1.04 (0.97–1.12)	0.266	1.12 (1.02–1.22)	0.013*
Teaching sessions	Ref	0.96 (0.91–1.01)	0.134	1.02 (0.95–1.10)	0.503
Number of students in class	Ref	1.01 (1.00–1.01)	0.023*	1.00 (0.99–1.01)	0.844
Number of breaks	Ref	0.99 (0.30–3.38)	0.999	0.23 (0.04–1.32)	0.099
Safety training at work (Yes)	Ref	0.75 (0.30–1.85)	0.535	0.23 (0.05–1.12)	0.069
Physical activity (inadequate)	Ref	4.29 (0.89–20.58)	0.069	0.83 (0.20–3.53)	0.801
Extracurricular activities (Yes)	Ref	0.69 (0.33–1.44)	0.323	0.59 (0.22–1.55)	0.281
Hosmer–Lemeshow test (*p*-value)	0.4103				

OR, odds ratio; CI, confidence interval; MSDs, musculoskeletal disorders; p50, 50th percentile (p50 = 2 body sites); Ref, reference category. *p-value was significant.

### Association between MSDs in individual body site and occupational factors

Regression analysis for MSDs in specific body was done for all body sites, however, the Hosmer–Lemeshow test only supported the models for the neck (*p* = 0.090) and low back pain (*p* = 0.516). The neck model showed that there was a significant positive association between neck MSDs and age (OR = 1.02, *p* = 0.016), working hours (OR = 1.07, *p* = 0.037), and number of classes (OR = 1.07, *p* = 0.039). Interestingly, there was a significant negative association between neck MSDs and number of students in a class (OR = 0.99, *p* = 0.043). For MSDs at the lower back, there was a significant positive association between low back MSDs and BMI (OR = 1.03, *p* < 0.001). Supplementary Table S1 provides the details of the variables selected for each body site model using backward elimination method, and corresponding odds rations with their respective 95% confidence intervals.

## Discussion

Work-related musculoskeletal disorders (WMSDs) lead to pain, functional dependency, and decreased quality of life, imposing a substantial burden on workers in different fields (26). Direct consequences of WMSDs include absenteeism, reduced productivity, increased healthcare costs, psychological distress, and early retirement (27). Our findings showed that over half of the study participants had MSDs at one or more body sites, females being more affected compared to males. Multiple administrative, ergonomic and lifestyle factors significantly influenced MSDs among teachers.

In the current study, the 12-month prevalence of MSDs among primary and secondary school teachers was 61.5%. This finding is comparable with the findings reported by Durmus and Ilhanli (60.3%) in Turkey (28). Despite the differences in geographical and socioeconomic settings, both studies involved primary school teachers, who are likely to have higher workloads, and whose teaching activities involve multiple repetitive tasks, and static working positions. On the contrary, other studies had demonstrated higher prevalence of MSDs among teachers elsewhere. For example, Kraemer et al. (6) reported that all participants experiences MSDs at one or more body sites, while Cheng et al. (15), and Althomali (16) both found prevalence of over 90%. This large difference noted here could be because the participants were from public schools that usually train more students that could be beyond recommended teacher-to-student ratio, thus subjecting teachers to heavy workload. Also, other teachers were from elementary level where they have to individualize teaching activities based on the need of each student, an overwhelming duty that is likely to cause over exhaustion. On the other hand, the prevalence of less than 50% was reported by Alias et al. (29) and Karakaya et al. (20) in Malaysia and Turkey, respectively. Perhaps significant difference in improved infrastructure, working conditions, and education and health systems compared to Tanzania could be the reason for reduced prevalence. In addition, we found that the proportion of MSDs was high in females than in males. This finding is supported by previous studies that showed that MSDs were significantly higher in female gender than in men (30–32). Perhaps this is because, physiologically, women are more at risk of low muscle mass, early degeneration that may contribute to MSDs.

We found that the most affected part both for the past 7 days and 12 months was the lower back followed by the neck. On the contrary, in Malaysia it was found that the most affected body parts for the past 7 days and 12 months were the feet and knees (29). Also, in Cameroon, the 12-month and seven-day prevalence was higher in the neck followed by the lower back and shoulders (19). Additionally, Ojukwu et al. (18) reported that shoulder and neck had the highest prevalence. The differences observed could perhaps be because of the setup of working environment. For instance, teachers who are subjected to standing for longer periods during teaching, and have many class sessions per week are likely to complain of discomfort at their feet and knees. Likewise, teachers working in prolonged sitting position are at greater risks of developing back and neck pain. Teachers repeatedly writing on the boards, particularly overhead, have higher chances of perceiving ache at the shoulders and neck. Even though teachers all over the world are engaged in nearly similar teaching activities, specific body part may develop MSDs depending on nature of facilities at work, infrastructures, ergonomics knowledge and other lifestyle factors (5).

The current study shows that there was a significant positive association between 12-month prevalence of MSDs and working hours in a day. A similar finding was reported in Taiwan, showing that teachers working for more than recommended duration per week were at higher risks of developing MSDs (15). The probable reason could be because long hours of working expose teachers to exhaustion, prolonged static and awkward positions, or repetitive tasks that are directly associated with MSDs. Also, we found significant positive associations between MSDs in the last 12 months and work duration and number of classes. This observation corresponds to a study by Fahmy et al. (7) that also found that the number of students per classroom and the number of classes per week were positively associated with WMSDs among teachers. The similarity of this finding could be because most teachers across the globe tend to have more workloads. Besides, high number of class sessions and weekly teaching hours increase both physical and mental demands of the job leading to greater risks for MSDs. In addition, we found a strong positive association between multisite MSDs and BMI for the past 12 months. A finding similar to a previous study (33) that showed a significant relationship between BMI and musculoskeletal symptoms in the working population. This relationship could perhaps be due to the fact that high BMI may subject the more mechanical stress to the joints and spine leading to discomfort, ache or pain.

Our study also found that, in addition to BMI, MSDs over the last 7 days was positively associated with age, gender, and number of students. In the current study, age was significantly associated with MSDs at more than two body sites. This finding corresponds to finding from a survival analysis study which showed that biological aging contributed to multiple MSDs that may affect multiple body sites (34). Consequently, a recent study from Nigeria has shown signification difference between males and females for MSDs in favor of males (35). This could be supported by biological factors like lower muscle mass and fluctuations of hormones that influence bone resorption. Also, research shows that high number of students is likely to increase workload for teachers posing a major risk for MSDs (36). This finding from previous study supports our findings that showed that neck pain was positively associated with working hours and number of classes. This conforms to a previous report that awkward postures of the neck and repetitive neck movements are likely increase the risks of MSDs (37).

This study has provided insights on the extent of musculoskeletal disorders among primary and secondary school teachers in a low-resourced country. It was discovered that having MSDs at any part of the body result to difficulties in carrying out normal duties at work. Understanding the magnitude of the MSDs and their associated factors in teaching profession is important in planning respective preventive interventions. The key strengths of our study include the use of multi-stage sampling technique that ensured that every teacher in the municipality had a chance to participate in the study, thus reducing bias. Also, we recruited a relatively representable sample accordingly. However, our study had several limitations: this study being a descriptive cross-sectional study, it could not ascertain the causational relationship as MSDs could be caused by other confounders that were not measured. Nevertheless, the analysis involved fitting regression model that considered both crude and adjusted odds ratios to estimate the associations. Further, the use of a questionnaire that required information from the past 12 months could influence responses to recall bias, however, all participants were given enough time to fill the questionnaire truthfully and correctly as possibly they could. Moreover, gathering data from only teachers who were present on the day of data collection could have subjected out study to selection bias. Also, considering the multifactorial causes of MSDs, such as poor sleep quality and stress, which were not evaluated in this study, affects our impression on the factors observed to influence MSDs. Finally, collecting data from one municipal council may not be generalized across the nation, nonetheless, most municipalities have nearly the same socioeconomic characteristics.

## Conclusion

Over 50 % of primary and secondary school teachers in Moshi municipal reported having MSDs at one or more body sites. The most commonly affected areas were the neck, lower and upper back. Occupational factors such as long working hours, high number of classes, number of students in class, and duration of work increased the risk of MSDs among teachers. Additionally, BMI, gender and age were positively associated with MSDs at various body parts. Adhering to recommended classes per week, teacher-pupil ratio, and working hours may help to prevent the MSDs among teachers. Also, active living could be an essential strategy of preventing MSDs among teachers as it plays crucial part in weight management.

## Data Availability

The raw data supporting the conclusions of this article will be made available by the authors, without undue reservation.
