# Supplementary material for: Prevalence and factors associated with musculoskeletal disorders among primary and secondary school teachers
Source: Front Public Health. 2025 Oct 28;13:1654131. doi: 10.3389/fpubh.2025.1654131 (PMC12602518; doi:10.3389/fpubh.2025.1654131)
Supplement: Supplementary file 1 [file Table_1.pdf]

Supplementary Table S1. Factors associated with MSDs in individual body site

Variables	Neck	Shoulders	Elbows	Wrists	Upper back	Lower back	Hips	Knees	Ankles
	**OR (95% CI)**	**OR (95% CI)**	**OR (95% CI)**	**OR (95% CI)**	**OR (95% CI)**	**OR (95% CI)**	**OR (95% CI)**	**OR (95% CI)**	**OR (95% CI)**
Age	1.02 (1.00 - 1.04)*	1.00 (0.99 - 1.02)		1.01 (0.99 - 1.01)		1.01 (0.99 - 1.02)	0.99(0.98 - 1.00)		1.00 (0.99 - 1.01)
Gender (Female)		1.12 (0.99 - 1.26)		1.11 (1.00 - 1.24)*	1.16 (1.01 - 1.32)*	1.07 (0.92 - 1.24)		0.95 (0.86 - 1.06)	1.04 (0.94 - 1.15)
BMI	1.01 (0.99 - 1.02)	0.99 (0.98 - 1.00)			1.01 (0.99 - 1.02)	1.03 (1.01 - 1.04)***		1.00 (0.99 - 1.01)	1.01 (1.00 - 1.02)*
Alcohol drinking (Yes)		1.13 (0.99 - 1.29)	0.95 (0.86 - 1.05)	1.04 (0.92 - 1.17)					
Smoking status (Yes)			1.12 (0.88 - 1.43)	1.23 (0.92 - 1.65)	1.21 (0.86 - 1.70)	1.41 (0.97 - 2.04)		0.86 (0.66 - 1.12)	0.88 (0.69 - 1.13)
Marital status									
Married				0.66 (0.40 - 1.10)	0.68 (0.37 - 1.24)				
Single				0.75 (0.45 - 1.27)	0.72 (0.38 - 1.34)				
Widowed				0.76 (0.42 - 1.35)	0.96 (0.48 - 1.92)				
Education level									
Degree									0.94 (0.81 - 1.09)
Diploma									0.83 (0.71 - 0.98)*
Master									1.01 (0.81 - 1.25)
Commuting distance			0.99 (0.99 - 1.00)			0.99 (0.98 - 1.00)		0.99 (0.99 - 1.00)	
Work duration	0.98 (0.96 - 1.00)	1.01 (0.99 - 1.02)	1.01 (1.00 - 1.01)**				1.01 (0.99 - 1.02)	1.01 (1.00 - 1.02)**	
Work hours	1.07 (1.00 - 1.15)*		0.96 (0.92 - 1.00)		0.97 (0.91 - 1.04)	0.97 (0.91 - 1.04)	0.95 (0.91 - 0.99)*	1.02 (0.97 - 1.07)	1.02 (0.98 - 1.07)

Number of classes	1.07 (1.00 - 1.14)*		1.04 (1.00 - 1.08)*	1.03 (0.99 - 1.08)	1.05 (0.99 - 1.12)		1.02 (0.98 - 1.06)	0.98 (0.94 - 1.02)	1.07 (1.03 - 1.11)***
Teaching sessions	1.01 (0.99 - 1.02)	1.00 (0.99 - 1.01)	1.00 (0.99 - 1.01)	0.99 (0.99 - 1.00)		0.99 (0.99 - 1.01)	1.00 (0.99 - 1.01)		0.99 (0.99 - 1.00)
Number of students	0.99 (0.99 - 1.00)*				0.99 (0.99 - 1.00)		0.99 (0.99 - 1.00)		
Number of breaks			0.94 (0.81 - 1.10)					0.92 (0.78 - 1.09)	0.88 (0.76 - 1.03)
Work position									
Sitting	0.98 (0.74 - 1.31)		1.19 (0.99 - 1.44)				1.23 (1.03 - 1.47)*		
Standing	0.89 (0.77 - 1.02)		1.01 (0.92 - 1.11)				0.98 (0.90 - 1.06)		
Walking	1.37 (0.98 - 1.91)		1.27 (1.00 - 1.56)*				1.08 (0.87 - 1.32)		
Type of chair (without support)	0.87 (0.72 - 1.05)	0.94 (0.80 - 1.11)				1.06 (0.87 - 1.29)	1.08 (0.96 - 1.21)	0.94(0.82 - 1.08)	0.91 (0.79 - 1.04)
Safety training (Yes)	0.90 (0.76 - 1.05)	0.89 (0.78 - 1.03		0.97 (0.85 - 1.10)	0.87 (0.74 - 1.01)		1.16 (1.05 - 1.28)**		
Presence of assistive teaching devices (Yes)		1.05 (0.94 - 1.18)		1.18 (1.06 - 1.31)**	1.19 (1.05 - 1.36)**	0.91 (0.79 - 1.05)			
Engagement in extracurricular activities (Yes)		0.92 (0.82 - 1.03)		0.94 (0.84 - 1.04)	1.04 (0.92 - 1.18)				
Physical activity (Inadequate)			1.07 (0.94 - 1.23)			1.14 (0.93 - 1.41)	1.06 (0.94 - 1.19)	1.13 (0.97 - 1.31)	
**Hosmer_Lemeshow test (p-value)**	**0.08953**					**0.5156**			

Abbreviations: BMI, Body Mass Index; CI, Confidence interval; OR, odds ratio

* p < 0.05

** p < 0.01

*** p < 0.001
